# Determinants of temporal change in telomere length and its associations with chronic complications and mortality in type 2 diabetes: the Fremantle diabetes study phase II

**DOI:** 10.1186/s12933-025-02832-3

**Published:** 2025-07-03

**Authors:** Chieh-Hsin Yang, Michael L H Huang, Wendy A Davis, Alicia J Jenkins, Timothy M E Davis

**Affiliations:** 1 Baker Heart and Diabetes Institute, 75 Commercial Road, Melbourne, VIC 3004 Australia; 2 Baker Department of Cardiometabolic Health, University of Melbourne, Melbourne, VIC Australia; 3 NHMRC Clinical Trials Centre, University of Sydney, Sydney, NSW Australia; 4 Faculty of Medicine Nursing and Health Sciences, Monash University, Melbourne, VIC Australia; 5 Medical School, University of Western Australia, Fremantle Hospital, P. O. Box 480, Fremantle, WA 6959 Australia; 6 Department of Endocrinology and Diabetes, Fiona Stanley and Fremantle Hospitals Group, Murdoch, WA Australia

**Keywords:** Telomere length, Cardiovascular disease, Diabetes complications, Type 2 diabetes, Mortality

## Abstract

**Background:**

Relative telomere length (rTL), a biomarker of biological ageing, has been implicated in type 2 diabetes and its complications. We aimed to identify the associates of rTL change over 4 years (∆rTL), and to investigate whether rTL and ∆rTL are associated with complications and mortality in adults with type 2 diabetes from the Australian observational community-based Fremantle Diabetes Study Phase II (FDS2).

**Methods:**

Participants (n = 819) from the FDS2 cohort had baseline and Year-4 (mean ± SD 4.2 ± 0.4 years) rTL measured by qPCR (intra- and inter-assay %CV: 0.56% and 2.69%, respectively). The rTL change (∆rTL; % change/year) was categorised as Shortened (< − 2.69%), Unchanged (− 2.69% to + 2.69%) or Lengthened (> + 2.69%). Multiple logistic regression identified clinical and biochemical determinants of ∆rTL Shortened versus Not Shortened (Unchanged plus Lengthened). rTL and ∆rTL (continuous and categorical) were added to Cox and competing risk regression models of conventional predictors of major complications, CVD death and all-cause mortality during a mean ± SD 11.5 ± 2.1 years of follow-up.

**Results:**

rTL was inversely correlated with age (*r* = − 0.186, *P* < 0.001). ∆rTL was shortened in 25.5% subjects, unchanged in 10.5%, and lengthened in 64.0%. Shortening was associated with older age, male sex, smoking, obesity, lipid-modifying drug use, and higher platelet count and serum bilirubin levels (*P* < 0.05). There were no statistically significant unadjusted or age- and sex-adjusted associations between baseline rTL, Year-4 rTL, or ∆rTL, and any incident micro- or macrovascular complications. In unadjusted Cox regression, ∆rTL lengthening was associated with a lower risk of CVD death (hazard ratio 0.98 (0.97, 0.99), *P* = 0.042) but this association became non-significant after adjustment for conventional risk factors.

**Conclusions:**

In adults with type 2 diabetes, rTL does not always shorten over time. rTL and ∆rTL were associated with baseline conventional cardiometabolic risk factors but not independently with major incident complications. There was a weak association between ∆rTL and CVD mortality. These findings question the utility of rTL and ∆rTL in usual type 2 diabetes care.

**Supplementary Information:**

The online version contains supplementary material available at 10.1186/s12933-025-02832-3.

## Background

Diabetes is associated with chronic complications and consequently increased morbidity and mortality [1]. Because there is a residual risk of complications even when traditional modifiable risk factors are well controlled, novel biomarkers could assist in the early identification of people susceptible to adverse outcomes, and facilitate the development of new treatment targets and monitoring tools [2]. One such biomarker is telomere length which has been implicated in age-related diseases including cardiovascular disease (CVD) [3] and dysglycaemia [4]. Telomeres are protective repeating DNA sequences on the ends of chromosomes that maintain genome integrity. Telomeric DNA shortens during mitotic cycles until a critical telomere length is reached, at which time cells enter senescence and apoptosis [5]. Nevertheless, telomere regulation exhibits significant plasticity and is known to be modulated by both inherited and acquired factors [6, 7].

People with type 2 diabetes have shorter telomeres than age-matched individuals without diabetes, consistent with premature ageing [8]. This may reflect increased oxidative stress and systemic inflammation which can promote telomeric DNA damage and subsequent telomere shortening [9]. In addition, there is some evidence that the activity of telomerase, an enzyme which adds telomeric repeats to chromosome ends, may be attenuated in diabetes [10] and this has been linked to dysglycaemia in preclinical studies [11, 12]. In epidemiological studies of people with type 2 diabetes, baseline telomere length was independently associated with chronic diabetes complications [13, 14]. However, these latter studies analysed telomere length at only one time-point despite its potential modulation by modifiable environmental factors [7]. Indeed, in longitudinal studies of people with type 1 diabetes [15] or impaired glucose tolerance (IGT) [16], 40–66% of participants exhibited telomere lengthening rather than shortening during follow-up. In the context of type 2 diabetes, the blood glucose-lowering medications metformin [17] and sitagliptin [18], as well as CVD risk factor treatments including calcium channel blockers, angiotensin receptor blockers (ARBs) and statins [19, 20], appear to be telomere protective. These various considerations strongly suggest that there is a potentially dynamic relationship between telomere length and both clinical management and complications in type 2 diabetes that merits exploration in longitudinal studies. Furthermore, there are no studies that have examined the relationship between changes in telomere length and mortality.

To address these knowledge gaps, we have utilised samples and data from adults with type 2 diabetes from the community-based observational Fremantle Diabetes Study Phase II (FDS2) to explore (i) the nature and determinants of the temporal change in telomere length, and (ii) its association with chronic diabetes complications and mortality.

## Methods

### Participants

The FDS2 is a cohort study conducted in a postcode-defined urban community of 157,000 residents in Western Australia (WA). Details of recruitment, participant characteristics and study procedures have been reported previously [21]. Participants (n = 1732) were recruited between 2008 and 2011, including 1482 (85.6%) with type 2 diabetes after excluding those with Maturity Onset Diabetes of the Young or Latent Autoimmune Diabetes of Adults [22]. The present sub-study included those FDS2 participants with type 2 diabetes who attended scheduled assessments at two timepoints a mean ± SD 4.2 ± 0.4 years apart. The FDS2 was approved by the Southern Metropolitan Area Health Service Human Research Ethics Committee and conducted in accordance with the principles of the Declaration of Helsinki. All subjects gave written informed consent.

### Clinical and laboratory methods

Baseline assessments included a comprehensive questionnaire, physical examination and collection of fasting blood and urine samples. Each participant was invited to attend biennial reviews over six years post-recruitment and to complete biennial clinical questionnaires between face-to-face assessments. Racial/ethnic background was categorised based on self-selection, country/countries of birth and parents’/grandparents’ birth, and language(s) spoken at home as Anglo-Celt, Southern European, Other European, Asian, Aboriginal or mixed/other. Central obesity was determined using body mass index (BMI), and a body shape index (ABSI) which is a more reliable estimate of visceral adiposity [23]. HbA_1c_, serum glucose, urea, electrolytes, lipids, and uric acid, liver function, and urine albumin and creatinine were measured using standard automated methods in a single nationally accredited laboratory. Baseline serum C-reactive protein was measured on an Architect ci16200 analyser using a high-sensitivity protocol (hsCRP; Abbott Diagnostics Australia, North Ryde, NSW, Australia). Serum alpha-2 macroglobulin and hyaluronic acid were measured on a Cobas 501 analyser (Roche Diagnostics Australia, North Ryde, NSW, Australia).

### Ascertainment of complications and mortality

Chronic complications were ascertained using standard criteria [21]. Retinopathy was assessed from colour fundus photography. A urinary albumin:creatinine ratio (uACR) was determined from a first-morning sample and an eGFR was calculated [24]. Distal symmetrical polyneuropathy (DSPN) was defined using the vibration perception threshold [25]. Peripheral arterial disease (PAD) was defined as an ankle brachial index ≤ 0.90 or a diabetes-related lower extremity amputation (LEA). Coronary heart disease comprised myocardial infarction, angina, coronary artery bypass grafting or angioplasty. Cerebrovascular disease was defined as a stroke and/or transient ischaemic attack.

The Hospital Morbidity Data Collection (HMDC) contains validated information for all public/private hospitalisations in WA since 1970 and the Death Register contains data on all deaths [26]. The FDS2 database was linked to these databases through the WA Data Linkage System (WADLS), as approved by the WA Department of Health Human Research Ethics Committee. HMDC data supplemented that from FDS assessments of prior/prevalent complications at baseline. Incident cerebrovascular disease and ischaemic heart disease were determined using WADLS data. Three-point major adverse cardiovascular events (MACE) was defined as non-fatal myocardial infarction (MI), stroke, or death from cardiac/cerebrovascular causes or sudden death. CVD mortality and all-cause death were determined through death register linkage between entry and end-2021. HMDC data were used to calculate the Charlson Comorbidity Index (CCI) [27] excluding conditions coded as diabetes-specific.

### Telomere length measurement

Relative telomere length (rTL), a measurement of telomere length normalised to a reference sample, was determined at baseline and Year-4, and the rTL change (∆rTL) per year calculated as described previously [28] and briefly outlined below. Peripheral blood-derived DNA was used due to its availability and positive correlations between telomere length in leucocytes and most solid tissues [29]. DNA was extracted from whole blood (QIAamp DNA Blood Mini kit, Qiagen, Hilden, Germany) and stored at − 80 °C until analysis. DNA concentration and quality (260 nm/280 nm) were determined in duplicate (Nanodrop 2000c spectrophotometer; Thermo Fisher Scientific, Waltham, MA, USA). Samples with DNA concentration < 12.5 ng/mL (n = 111) were concentrated using a rotary sample concentrator (RVC 2–18, Martin Christ, Osterode am Harz, Germany) at 65 °C for 5-h. DNA samples (n = 1894; 947 FDS2 participants at each timepoint) were diluted in Ultrapure distilled water (Thermo Fisher Scientific) to 12.5 ng/mL using an automatic liquid handler (epMotion 5075t with epBlue software v4.09, Eppendorf, Hamburg, Germany). Twenty-two (2.29%) participants were excluded due to poor DNA yield at Baseline or Year-4 (n = 13; 1.35%) or due to failed rTL quality check (n = 9; 0.95%).

rTL was determined by quantitative polymerase chain reaction (qPCR) using 25 ng DNA [28] adapted for 384-well plate using a QuantStudio 7 Flex qPCR system (Thermo Fisher Scientific). rTL was calculated using the ∆∆Ct method: telomere and the single copy gene human β-globin (*HBG*) relative to a reference human DNA sample (“QC”) for normalisation. This QC was also used in previous studies employing the same rTL protocol [28] to facilitate comparisons across more than five independent cohorts [4, 14, 15, 30–32]. Briefly, for each qPCR plate and FDS2 or QC sample, telomere qPCR Ct was subtracted from HBG Ct to obtain ΔCt _(FDS2)_ and ΔCt _(QC)_. Then, ΔCt _(QC)_ was subtracted from ΔCt _(FDS2)_ to obtain a rTL value for Baseline and Year-4. Primers used were telomere (Telomere A: 5’-CGGTTTGTTTGGGTTTGGGTTTGGGTTTGGGTTTGGGTT-3’; Telomere B: 5’-GGCTTGCCTTACCCTTACCCTTACCCTTACCCTTACCCT-3’) and *HBG* (*hbg*1: 5’-GCTTCTGACACAACTGTGTTCACTAGC-3’; *hbg*2: 5’- CACCAACTTCATCCACGTTCACC-3’) (Thermo Fisher Scientific). An automatic liquid handler epMotion 5075t (Eppendorf) was employed for qPCR reaction preparations to increase accuracy. A no template control of ultrapure distilled water (Thermo Fisher Scientific) and QC were included in each qPCR plate for normalisation of any plate-to-plate variability and ΔΔCt calculation. Baseline and Year-4 samples from each subject were assayed on the same plate in triplicate. Only samples with replicate CV < 2.5% were accepted, otherwise the mean of the two closest replicates < 0.5 Ct apart were used. ΔrTL between the two timepoints were computed as % change per year using the previous reported formula ${(2}^{\left(\frac{\left(Year-4 rTL - Baseline rTL\right)}{t}\right)}-1)$×100%, where *t* is years between timepoints [15].

Intra-assay %CV was 0.58% for rTL and 0.69% for *HBG*. Inter-assay %CV of the rTL and *HBG* were 2.69% and 1.46%, respectively. Thus, changes beyond the technical variability of 2.69% were deemed biological meaningful. Telomeres which increased or decreased by < 2.69% between the two timepoints were considered unchanged. Relative to the QC sample, a negative ΔΔCt rTL indicates shorter telomeres and a positive ΔΔCt rTL indicates longer telomeres. For ΔrTL, a positive value indicates lengthened telomeres and a negative value reflects shortened telomeres. ΔrTL was normally distributed (mean ± SD; 8.4 ± 21.0%; median 8.4%; range − 93.8% to 115.7%). ΔrTL was categorised as Shortened (< − 2.69%), Unchanged (± 2.69%), or Lengthened (> 2.69%).

### Statistical analyses

Data are reported as percentage, mean ± SD, geometric mean (SD range), or, when variables are not approximately normally distributed, median [interquartile range]. Two-way comparisons used Fisher’s or Fisher-Freeman-Halton exact tests for independent samples, Student’s *t*-test for approximately normally distributed variables, and Mann–Whitney U-test for non-normally distributed variables. Linear regression was used to investigate individual associations between ΔrTL as continuous variable and baseline variables of interest with and without adjustment for age and sex. Multiple logistic regression with backward stepwise modelling (entry *P* ≤ 0.049, removal *P* ≥ 0.050) of plausible variables with bivariable *P* < 0.020 was used to identify independent clinical and biochemical determinants of ∆rTL shortening vs not shortening after adjusting for age, sex and baseline rTL. Multiple linear regression with backward stepwise modelling (entry *P* ≤ 0.049, removal *P* ≥ 0.050) of plausible variables with bivariable *P* < 0.020 (one variable removed at a time, the least significant first) was used to identify independent associates of ΔrTL (continuous).

For five-year incident complications, end-stage renal disease (ESRD), three-point MACE, and mortality to end-2021, Cox regression was conducted with baseline rTL, Year-4 rTL and ΔrTL as independent variables (separately), unadjusted and adjusted for age and sex. For four-year incident complications, logistic regression was used with baseline rTL, Year-4 rTL and ΔrTL as independent variables (separately), unadjusted and adjusted for age and sex. Participants with prior events associated with the respective complications were excluded from the analyses related to incident complications. Since all participants were alive at Year-4, there was no risk of immortal time bias, so both baseline and Year-4 rTL and ΔrTL (as continuous or categorical variables) were added separately to the most parsimonious Cox regression models of determinants of all-cause and CVD mortality. Competing risk regression analysis of CVD mortality adjusted for the competing risk of death from non-CVD causes was performed. A sensitivity analysis was undertaken for Year-4 rTL and ΔrTL, in which Year-4 was used as baseline for the Cox and competing risk models of time to CVD mortality and Year-4 variables were considered for entry. Since few baseline data were missing, available case analyses were performed. A two-tailed significance level of *P* < 0.050 was used throughout.

## Results

### Participant characteristics

There were 819 participants with rTL measurements at baseline and Year-4 (mean ± SD 4.2 ± 0.4 years apart). Compared with the 663 FDS2 participants with type 2 diabetes who were not studied, the included cohort was not significantly different in age at entry (65.8 ± 12.8 vs. 65.7 ± 10.4 years, *P* = 0.798) or sex (48.9% vs. 53.7% male, *P* = 0.067) but their diabetes duration was shorter (10.0 [3.0–17.0] vs. 8.0 [2.0–15.0] years, *P* < 0.001). Characteristics of the 819 FDS2 participants at baseline and Year-4 are summarised in Table 1. Compared to baseline, there were significant increases in fasting glucose and HbA_1c_ despite intensification of glycaemic management, and in dyslipidaemia (lower serum HDL-cholesterol/raised triglycerides) despite increased use of lipid-modifying medication (predominantly statins). Systolic and diastolic blood pressures were lower in association with increased antihypertensive drug use including angiotensin converting enzyme inhibitors (ACEi)/ARBs. uACR did not change but eGFR declined. Current smoking and central obesity decreased. Macrovascular (cerebrovascular disease, coronary heart disease, PAD) and microvascular (DSPN, any retinopathy) complications increased. Baseline rTL inversely correlated with age (*r* = ‒0.186, *P* < 0.001).

**Table 1:** Baseline and Year-4 characteristics of 819 FDS2 participants with type 2 diabetes who attended both assessments

	n^§^	Baseline	Year-4	*P*-value^^^
Age (years)	819	65.7 ± 10.4	69.9 ± 10.4	–
Male (%)	819	53.7		
Ethnic background (%):	819			
Anglo-Celt		58.9		
Southern European		11.4		
Other European		7.7		
Asian		4.4		
Aboriginal		2.3		
Mixed/other		15.4		
Age at diabetes diagnosis (years)	819	56.2 ± 11.4		
Diabetes duration (years)	819	8.0 [2.0–15.0]	12.2 [6.5–19.3]	–
Smoking status (%):	812			0.005
Never		43.0	43.0	
Ex		50.0	52.0	
Current		7.0	5.0	
Alcohol consumption (standard drinks^¶^/day)	748	0.1 [0–1.5]	0.1 [0–0.8]	< 0.001
Fasting serum glucose (mmol/L)	816	7.1 [6.1–8.5]	7.8 [6.4–9.4]	< 0.001
HbA_1c_ (%)	819	6.7 [6.2–7.5]	6.8 [6.2–7.6]	0.048
Diabetes treatment (%):	808			< 0.001
Diet		28.1	19.4	
Oral hypoglycaemic agents (OHAs)/non-insulin injectables		52.4	53.7	
Insulin alone		4.2	5.7	
Insulin ± OHAs/non-insulin injectables		15.3	21.2	
Metformin therapy (%)	807	62.8	69.9	< 0.001
BMI (kg/m^2^)	809	31.2 ± 5.7	31.2 ± 6.0	0.674
ABSI (m^11/6^ kg^−2/3^)	807	0.0811 ± 0.0049	0.0808 ± 0.0049	0.021
Central obesity^*^ (%)	812	71.6	67.0	< 0.001
Systolic blood pressure (mmHg)	816	145 ± 21	141 ± 20	< 0.001
Diastolic blood pressure (mmHg)	815	80 ± 12	77 ± 12	< 0.001
Antihypertensive therapy (%)	813	74.9	80.6	< 0.001
ACEi/ARB therapy (%)	813	66.4	70.8	0.006
Total serum cholesterol (mmol/L)	816	4.3 ± 1.2	4.3 ± 1.0	0.082
Serum HDL-cholesterol (mmol/L)	816	1.24 ± 0.32	1.15 ± 0.30	< 0.001
Serum triglycerides (mmol/L)	816	1.5 (0.9–2.4)	1.6 (0.9–2.6)	0.009
Lipid-lowering therapy (%)	813	70.7	75.0	0.006
Fibrate therapy (%)	813	2.3	4.8	< 0.001
hsCRP (mg/L)		2.2 (0.7–6.5)	Not measured	–
Aspirin therapy (%)	811	38.6	39.1	0.815
Cerebrovascular disease (%)	819	7.0	7.7	0.031
Coronary heart disease (%)	819	25.8	32.6	< 0.001
PAD (%)	806	19.2	34.2^†^	< 0.001
DSPN (%)	812	36.1	53.9^‡^	< 0.001
eGFR category (%):	814			< 0.001
≥ 90 mL/min/1.73m^2^		38.8	19.4	
60–89 mL/min/1.73m^2^		47.7	58.8	
45–59 mL/min/1.73m^2^		7.9	11.2	
30–44 mL/min/1.73m^2^		4.4	6.9	
< 30 mL/min/1.73m^2^		1.2	3.7	
eGFR (mL/min/1.73m^2^)	814	81 ± 19	74 ± 20	< 0.001
uACR (mg/mmol)	799	2.8 (0.9–9.3)	2.9 (0.7–12.2)	0.584
Any retinopathy (%)	719	33.1	37.7^#^	< 0.001
Platelet count (× 10^9^/L)		246 (189–320)	Not measured	–
Serum albumin (g/L)	809	44 ± 3	42 ± 3	< 0.001
Serum Ƴ-glutamyl transferase (U/L)	809	31 (16–60)	31 (15–65)	0.109
Serum bilirubin (mmol/L)	794	9.9 (6.7–14.7)	10.1 (6.4–16.1)	0.348
Serum α-2 macroglobulin (g/L)		2.05 (1.45–2.89)	Not measured	–

^^^*P*-values are for paired comparisons; ^¶^1 standard drink = 10 g pure alcohol; ^*^waist circumference ≥ 90 cm in male or ≥ 85 cm in female; ^†^cumulative (i.e. PAD present at any of baseline, Year-2 and Year-4 assessments); ^‡^cumulative (DSPN present at any of baseline, Year-2 and Year-4 assessments); ^#^cumulative (i.e. any retinopathy present at any of baseline, Year-2 and Year-4 assessments). ^§^number of participants for whom data are available at both baseline and Year-4

### Baseline variables associated with ∆rTL

In unadjusted linear regression with ∆rTL as a continuous variable, male sex was associated with ∆rTL shortening (*P* < 0.001) but European (excluding Southern European) ethnicity was protective (Table 2). ΔrTL was negatively associated with central obesity by waist circumference and total serum cholesterol. When changes in variables of interest between baseline and Year-4 were considered, cessation of fibrate therapy was negatively correlated with ∆rTL, while change in total serum cholesterol levels was positively correlated (Additional file 1). In multiple linear regression analysis adjusted for age, sex, and baseline rTL, independent determinants of ∆rTL comprised European (excluding Southern European) ethnicity (positively), central obesity (negatively), serum HDL-cholesterol (negatively), longer follow-up (negatively), and fibrate cessation (negatively) (Table 3).

**Table 2:** Individual associations of baseline variables with ∆rTL as a continuous variable in people with type 2 diabetes from FDS2

	Unadjusted regression coefficient	*P*-value	*P*-value^*^
Age (years)	− 0.061	0.384	0.363
Male	− 4.722	0.001	< 0.001
Ethnic background:			
Anglo-Celt	− 1.770	0.236	0.149
Southern European	0.214	0.926	0.776
Other European	5.930	0.031	0.024
Asian	1.055	0.769	0.739
Aboriginal	− 2.222	0.649	0.434
Mixed/other	− 0.061	0.976	0.868
Age at diabetes diagnosis (years)	0.015	0.812	0.298
Diabetes duration (years)	− 0.127	0.153	0.299
Smoking status:			
Never	1.850	0.213	0.790
Ex	− 2.174	0.139	0.589
Current	1.319	0.638	0.609
Alcohol consumption (standard drinks/day)	− 0.503	0.188	0.537
Fasting serum glucose (mmol/L)	− 0.033	0.911	0.935
HbA_1c_ (%)	− 0.109	0.848	0.846
Diabetes treatment:			
Diet	1.080	0.509	0.792
Oral hypoglycaemic agents (OHAs)/non-insulin injectables	− 0.215	0.884	0.905
Insulin ± OHAs/non-insulin injectables	− 1.052	0.571	0.654
Metformin use	− 0.914	0.548	0.739
BMI (kg/m^2^)	− 0.055	0.664	0.321
ABSI (increase of 0.001 m^11/6^ kg^−2/3^)	− 0.170	0.259	0.972
Central obesity (by waist circumference; %)	− 2.407	0.140	0.023
Systolic blood pressure (mmHg)	− 0.014	0.683	0.781
Diastolic blood pressure (mmHg)	− 0.007	0.909	0.619
Taking antihypertensive medication	− 0.599	0.723	0.928
Taking ACEi/ARB	− 0.003	0.999	0.828
Total serum cholesterol (mmol/L)	− 0.863	0.175	0.028
Serum HDL-cholesterol (mmol/L)	− 1.421	0.537	0.127
Ln(Serum triglycerides (mmol/L))^^^	− 0.815	0.594	0.539
Taking lipid-lowering medication	− 0.299	0.853	0.998
Taking fibrates	− 6.043	0.216	0.271
Ln(hsCRP (mg/L))^^^	− 0.355	0.600	0.210
On aspirin	− 0.751	0.619	0.809
Cerebrovascular disease	− 3.388	0.240	0.324
Coronary heart disease	− 1.224	0.462	0.836
Peripheral arterial disease	− 0.341	0.855	0.636
eGFR (CKD-EPI) category:			
≥ 90 ml/min/1.73m^2^	0.419	0.782	0.798
60–89 ml/min/1.73m^2^	0.317	0.830	0.575
45–59 ml/min/1.73m^2^	− 0.073	0.979	0.882
30–44 ml/min/1.73m^2^	− 4.325	0.228	0.295
< 30 ml/min/1.73m^2^	0.765	0.909	0.910
Ln(uACR (mg/mmol))^^^	− 0.092	0.880	0.961
Any retinopathy	− 0.282	0.856	0.844
Ln(Platelets (× 10^9^/L))^^^	− 2.394	0.395	0.056
Serum albumin (g/L)	− 0.020	0.940	0.853
Ln(Ƴ-glutamyl transferase (U/L))^^^	− 1.958	0.072	0.126
Ln(Bilirubin (mmol/L))^^^	1.771	0.346	0.091
Ln(α-2 macroglobulin (g/L))^^^	− 1.782	0.407	0.633

^*^Age- and sex-adjusted *P*-values derived from linear regression with ∆rTL as the independent variable; ^^^An increase of 1 in ln(x) equates to an increase of 2.72 in x

**Table 3:** Multiple linear regression of independent baseline associates of ∆rTL as continuous variable in people with type 2 diabetes

	Regression coefficient	*P*-value
European ethnic background (excluding Southern European)	6.530	0.003
Central obesity (by waist circumference)	− 2.893	0.025
Serum HDL-cholesterol (increase of 0.1 mmol/L)	− 0.544	0.010
Duration of follow-up (increase of 1 year)	− 5.197	0.003
Stopped fibrate	− 17.960	0.033

Backward stepwise selection from variables with bivariable *P* < 0.020, adjusted for age, sex and baseline rTL

∆rTL was Shortened in 25.5% (n = 209), Unchanged in 10.5% (n = 86) or Lengthened in 64.0% (n = 524; Additional file 2), with Not Shortened (i.e. Unchanged and Lengthened groups combined) in 74.5% (n = 610). ∆rTL was inversely correlated with baseline rTL (r = − 0.568, *P* < 0.001). Those whose telomeres subsequently Shortened or were Unchanged had a longer baseline rTL than the Lengthened group (Baseline rTL 0.211 ± 0.89 in Shortened, 0.046 ± 0.65 in Unchanged, and − 0.774 ± 0.83 in Lengthened, *P* < 0.001**;** Additional file 2).

When Shortened (∆rTL < − 2.69%) and Not Shortened (∆rTL ≥ − 2.69%) were compared, individuals with Shortened rTL were more likely to be male, ex-smokers, on lipid-modifying medications, had lower serum bilirubin, increased central obesity and ABSI versus the Not Shortened group (*P* ≤ 0.044; Additional file 3). After adjusting for age, sex and baseline rTL, rTL Shortening was independently associated with being an ex-smoker, on lipid-modifying medications, with higher central obesity and platelet counts, and lower serum bilirubin when compared with the Not Shortened group (Table 4).

**Table 4:** Multiple logistic regression of independent baseline associates of ΔrTL Shortened versus Not Shortened in people with type 2 diabetes

	Odds ratio (95% CI)	*P*-value
Smoking status: Ex-smoker	1.54 (1.04, 2.27)	0.029
Central obesity (by waist circumference)	1.57 (1.02, 2.43)	0.042
On lipid-modifying medications	1.83 (1.19, 2.80)	0.006
Ln(Platelets (× 10^9^/L))^^^	2.41 (1.12, 5.20)	0.025
Ln(Bilirubin (mmol/L))^^^	0.54 (0.33, 0.89)	0.016

^^^An increase of 1 in ln(x) equates to an increase of 2.72 in x. Multiple logistic regression with backward stepwise modelling (entry *P* ≤ 0.049, removal *P* ≥ 0.050) of variables with bivariable *P* < 0.020, adjusted for age, sex and baseline rTL

### Risk of incident complications and mortality by baseline rTL, Year-4 rTL and ΔrTL

Complications and deaths were ascertained from a mean ± SD 11.5 ± 2.1 years of follow-up. Complications were classified as (i) those ascertained at biennial in-person FDS2 assessments between baseline and Year-4 (eGFR decline > 30%, new chronic kidney disease (eGFR < 60 mL/min/1.73m^2^), progression from normoalbuminuria to micro-/macroalbuminuria, development of any retinopathy, development of DSPN, and new PAD), (ii) five-year incident complications from WADLS data (stroke, MI, and diabetes-related foot ulcer hospitalisation and LEA), (iii) ten-year incident end-stage renal disease (ESRD) after baseline, and iv) development of ESRD and MACE from baseline (for baseline rTL) or Year-4 (for Year-4 rTL and ∆rTL) to the end of follow-up. There were no statistically significant unadjusted or age- and sex-adjusted associations between baseline rTL, Year-4 rTL, or ∆rTL, and any incident complications (Table 5).

**Table 5:** Individual logistic or Cox regression analyses of incident complications and mortality by baseline and Year-4 rTL, and ∆rTL in people with type 2 diabetes

Baseline rTL				
4-year incident complications:	n events/N participants^§^	Unadjusted OR (95% CI)	*P*-value	*P*-value^*^
eGFR decline > 30%	65/814	1.10 (0.84, 1.44)	0.506	0.166
CKD (eGFR ≥ 60 to < 60 ml/min/1.73m^2^)	90/698	0.96 (0.76, 1.20)	0.697	0.379
Normo- to micro-/macroalbuminuria	134/492	1.07 (0.87, 1.31)	0.532	0.226
Any retinopathy	36/467	1.04 (0.73, 1.50)	0.813	0.683
DSPN	143/518	0.92 (0.76, 1.11)	0.395	0.811
PAD	124/652	1.04 (0.85, 1.28)	0.706	0.285
5-year incident complications (from baseline):	n events/N participants^§^	Unadjusted HR (95% CI)	*P*-value	*P*-value^*^
Stroke (non-fatal and fatal)	11/798	1.29 (0.67, 2.47)	0.441	0.378
Myocardial infarction (non-fatal and fatal)	25/767	1.35 (0.88, 2.07)	0.168	0.961
Diabetic foot ulcer hospitalisation	10/778	0.97 (0.51, 1.87)	0.933	0.763
Diabetes-related LEA	4/815	0.74 (0.29, 1.89)	0.529	0.551
10-year incident complications (from baseline):	n events/N participants^§^	Unadjusted HR (95% CI)	*P*-value	*P*-value^*^
End-stage renal disease	23/817	1.26 (0.81, 1.97)	0.312	0.158
To end-2021 (from baseline):	n events/N participants^§^	Unadjusted HR (95% CI)	*P*-value	*P*-value^*^
End-stage renal disease	32/817	1.11 (0.76, 1.63)	0.583	0.347
3-point MACE^^^	151/747	0.89 (0.76, 1.05)	0.186	0.796
CVD mortality	74/819	0.84 (0.67, 1.06)	0.147	0.900
All-cause mortality	213/819	0.87 (0.75, 1.00)	0.043	0.964
Year-4 rTL				
4-year incident complications:	n events/N participants^§^	Unadjusted OR (95% CI)	*P*-value	*P*-value^*^
eGFR decline > 30%	65/814	0.96 (0.80, 1.14)	0.599	0.880
CKD (eGFR ≥ 60 to < 60 ml/min/1.73m^2^)	90/698	1.01 (0.85, 1.20)	0.919	0.293
Normo- to micro-/macroalbuminuria	134/492	1.05 (0.90, 1.22)	0.527	0.305
Any retinopathy	36/467	1.05 (0.80, 1.38)	0.706	0.547
DSPN	143/518	0.86 (0.73, 1.00)	0.057	0.652
PAD	124/652	1.08 (0.90, 1.29)	0.402	0.190
To end-2021 (from Year-4):	n events/N participants^§^	Unadjusted HR (95% CI)	*P*-value	*P*-value^*^
End-stage renal disease	21/806	0.92 (0.73, 1.33)	0.922	0.865
3-point MACE^^^	130/726	0.93 (0.85, 1.01)	0.089	0.507
CVD mortality	74/819	0.88 (0.81, 0.96)	0.004	0.046
All-cause mortality	213/819	0.91 (0.86, 0.98)	0.008	0.277
∆rTL				
4-year incident complications:	n events/N participants^§^	Unadjusted HR (95% CI)	*P*-value	*P*-value^*^
eGFR decline > 30%	65/814	0.99 (0.98, 1.00)	0.166	0.237
CKD (eGFR ≥ 60 to < 60 ml/min/1.73m^2^)	90/698	1.00 (0.99, 1.01)	0.822	0.794
Normo- to micro-/macroalbuminuria	134/492	1.00 (0.99, 1.01)	0.833	0.793
Any retinopathy	36/467	1.00 (0.98, 1.01)	0.881	0.974
DSPN	143/518	0.99 (0.98, 1.00)	0.247	0.513
PAD	124/652	1.00 (0.99, 1.01)	0.761	0.804
To end-2021 (from Year-4):	n events/N participants^§^	Unadjusted HR (95% CI)	*P*-value	*P*-value^*^
End-stage renal disease	21/806	1.01 (0.99, 1.03)	0.533	0.526
3-point MACE^^^	130/726	0.99 (0.98, 1.00)	0.454	0.662
CVD mortality	74/819	0.98 (0.97, 0.99)	0.042	0.071
All-cause mortality	213/819	0.99 (0.98, 1.00)	0.138	0.217

For 4-year incident outcomes, the *P*-values were derived from logistic regression with baseline rTL as the independent variable, unadjusted and adjusted for age and sex. For 5-year and 10-year incident outcomes and mortality, the *P*-values were derived from Cox regression with baseline rTL or Year-4 rTL as the independent variable, unadjusted and adjusted for age and sex. ^§^Number of incident events/number of participants without the respective complication at baseline. ^*^Adjusted for age and sex. ^^^3-point MACE: non-fatal MI or stroke or death from cardiac or cerebrovascular causes or sudden death

Both baseline and Year-4 rTL were significantly shorter in 213 subjects who had died by end-2021 versus the 606 survivors (baseline − 0.55 ± 0.97 vs. − 0.40 ± 0.94, *P* = 0.043; Year-4: − 0.30 ± 1.11 vs. − 0.004 ± 1.32, *P* = 0.004; Additional file 4). ∆rTL did not differ significantly by survivor status (*P* = 0.078). For all-cause mortality, unadjusted Cox regression revealed that rTL at both baseline and Year-4 was associated with lower risk (HR (95% CI) 0.87 (0.75, 1.00) and 0.91 (0.86, 0.98), respectively, for an increase of 1 in rTL), but these associations became non-significant after age and sex adjustment (Table 5). ΔrTL was not associated with all-cause mortality. For CVD mortality by unadjusted Cox regression, Year-4 rTL and ΔrTL were associated with lower risk (HR (95% CI) 0.88 (0.81, 0.96) and 0.98 (0.97, 0.99), respectively, for an increase of 1 in rTL; Table 5). After age and sex adjustment, Year-4 rTL remained significantly negatively associated with CVD mortality.

In Cox regression analysis of time to CVD mortality with adjustment for conventional risk factors, addition of Year-4 rTL added modestly to the most parsimonious Cox model (cause-specific HR (csHR) (95% CI): 0.87 (0.76, 1.00) for an increase of 1; Table 6). Neither baseline rTL nor ∆rTL (continuous/categorical) added significantly to the most parsimonious conventional model. After adjusting for the competing risk of death from non-CVD causes, the association between Year-4 rTL and CVD mortality was strengthened (subdistribution HR (sdHR) (95% CI): 0.86 (0.76, 0.97), *P* = 0.015; Additional file 5). We confirmed these observations by replacing baseline variables with the equivalent Year-4 variables which were considered for entry into the Cox (Additional file 6) and competing risk (Additional file 7) regression models. The association between Year-4 rTL and CVD mortality remained (csHR (95% CI): 0.89 (0.81, 0.99) for an increase of 1, *P* = 0.036; sdHR (95% CI): 0.88 (0.80, 0.97) for an increase of 1, *P* = 0.013). In competing risk regression using Year-4 as baseline and Year-4 variables, ∆rTL was also independently associated with CVD mortality (sdHR (95% CI): 0.99 (0.98, 0.9997) for an increase of 1, *P* = 0.045). In Cox regression analysis of time to all-cause death, after the addition of baseline rTL, Year-4 rTL or ∆rTL (continuous/categorical) to conventional risk factors did not add significantly to the most parsimonious conventional model (*P* ≥ 0.259).

**Table 6:** Cox regression models of time to cardiovascular disease mortality with measures of rTL (added separately) as the main variable of interest. Data are cause-specific hazard ratios (csHR) and 95% confidence intervals (CI)

	csHR (95% CI)	*P*-value	csHR (95% CI)	*P*-value	csHR (95% CI)	*P*-value	csHR (95% CI)	*P*-value
Baseline rTL (increase of 1)	0.90 (0.70, 1.15)	0.411						
Year-4 rTL (increase of 1)			0.87 (0.76, 1.00)	0.050				
∆rTL (increase of 1)					0.99 (0.98, 1.00)	0.233		
∆rTL categories:								
Unchanged							1.00	
Shortened							1.22 (0.57, 2.63)	0.607
Lengthened							0.99 (0.48, 2.06)	0.979

All models are adjusted for age, insulin use, heart rate, ln(NT-proBNP), peripheral arterial disease, retinopathy, and Charson’s Comorbidity Index

## Discussion

Utilising rich phenotypic and outcome data from the FDS2, we found that telomeres did not shorten in almost three-quarters of participants with type 2 diabetes during four years of follow-up. There was no association between baseline rTL, Year-4 rTL or ∆rTL and a range of incident micro- and macrovascular complications of diabetes. In Cox and/or competing risk predictive models of CVD death, but not all-cause death, the inclusion of Year-4 rTL and ∆rTL added modestly to conventional risk factors. Although these data do not support the use of single and serial rTL measurements in the clinical management of type 2 diabetes, they provide insights into the changing nature of the epidemiology of diabetes and its complications.

Previous studies show that telomeres do not always shorten over time in people in the general population [15, 33–35], or with IGT [16] or type 1 diabetes [15]. In the context of dysglycaemia, telomere length was stable or increased in 45% of people with type 1 diabetes over a median of 6.8 years [15], and telomere elongation occurred over 4.5 years in two-thirds of overweight, middle-aged participants with IGT in both the intervention and control arms of the Finnish Diabetes Prevention Study [16]. This latter figure is similar to the 64% with rTL lengthening in the present study. In the Finnish Diabetes Prevention Study, telomere lengthening was attributed to improved lifestyle in both groups, as reported in other clinical contexts [36]. In the present FDS2 participants, central obesity assessed from ABSI and waist circumference was significantly lower at Year-4 versus baseline, strongly suggesting improved adherence to lifestyle measures. In addition, the proportions taking metformin, ACEi/ARBs, statins and fibrates increased, all agents considered to be telomere protective [17, 19, 20, 37, 38]. These data suggest that telomere length can shorten or lengthen in response to non-pharmacological and pharmacological factors in type 2 diabetes.

We found no significant associations between baseline rTL, Year-4 rTL, or ∆rTL, and a range of chronic complications and all-cause mortality after adjustment for age and sex. Only Year-4 rTL was significantly and negatively associated with CVD mortality after age and sex adjustment. In the case of baseline rTL, studies from the Hong Kong Diabetes Register of type 2 diabetes found modest inverse associations with incident CVD and nephropathy after adjustment [13, 14], but the cohort differed substantially from FDS2 participants with, for example, worse glycaemia, higher smoking rates and a much lower proportion taking ACEi/ARB. A UK outpatient clinic- rather than population-based study also found a relatively weak association between shorter telomere length and incident ischaemic heart disease [39]. The Hong Kong Diabetes Register study found an association between short telomere length and CVD and all-cause mortality [13] but, in a study of type 2 diabetes utilising National Health and Nutrition Examination Survey (NHANES) data, telomere lengthening was, by contrast, positively associated with CVD death [40]. In the present Cox and competing risk models, independent associations of Year-4 rTL and ∆rTL with CVD mortality were of borderline statistical significance and may not add substantive predictive power in risk models with more prominent variables such as age, sex, dyslipidaemia and hypertension. Our findings relating to complications and mortality could be due to the fact that telomeres in elderly participants with type 2 diabetes have reached a critical length [30, 40], and the presence of other major risk factors might mask an independent effect of ∆rTL.

Overall, these findings from FDS2 and antecedent studies question the value of a single or serial rTL measurement in identifying an individual with type 2 diabetes at risk of adverse outcomes, but the epidemiological context should be considered. Longitudinal data from the FDS [41] and other diabetes studies [42] show a significant decline in the incidence of cardiovascular complications over recent decades. It is possible that studies older than FDS2 and/or involving higher risk selected outpatients [13, 39, 40] may not have captured recent improvements in management of community-based people with type 2 diabetes that have impacted favourably on both rTL and CVD risk. An association between rTL and complications would be more difficult to identify in this situation.

Evidence of the validity of the present data is provided by both the variables associated with ∆rTL and analytical considerations. Regarding the associates of ∆rTL, whether as a continuous or categorical (Shortened versus Not Shortened) variable, we and others found that telomere attrition rate was substantially determined by age, sex, and baseline telomere length regardless of follow-up duration [15, 16, 33–35]. For ∆rTL as a continuous variable, the inverse associations with age, male sex and baseline rTL suggest that, in older males with a longer initial telomere length, ∆rTL was more likely to shorten. There was the same inverse relationship with duration of follow-up, and thus of diabetes, and for obesity assessed by waist circumference. Previous studies also found that obesity, a common comorbidity in type 2 diabetes which is also linked to increased oxidative stress and systemic inflammation, was a leading cause of telomere attrition [43, 44] and that weight reducing strategies such as caloric restriction or bioenteric intragastric balloon placement can maintain or even increase telomere length [45–48]. The strong positive relationship between ∆rTL and European ethnic background, excluding Anglo-Celts and Southern Europeans, seems at odds with evidence that these populations in Europe have a tendency to premature ageing [49]. However, as FDS2 participants are first- or second-generation migrants, they may be relatively healthy compared with residents in relatively socioeconomically deprived countries in, for example, Eastern Europe. The inverse association between baseline serum HDL-cholesterol and ∆rTL seems counterintuitive but has been reported previously in a general population study [50] and may relate to HDL dysfunction present in diabetes. In participants who stopped fibrate therapy, ∆rTL was likely to shorten, which is consistent with in vitro data [38].

In relation to ∆rTL categories, smoking increases oxidative stress [51] and has been associated with shorter telomeres in large studies [52, 53]. Our results showed that telomeres in ex-smokers were 1.54 times more susceptible to shortening over four years, in keeping with a study in type 1 diabetes in which current or ex-smoking status was inversely correlated with ΔrTL [15]. Consistent with analyses using ∆rTL as a continuous variable, central obesity was associated with > 50% increased risk of telomere shortening in our FDS2 participants. Lipid-modifying drugs were telomere-protective in an in vitro model of diabetes [38] and cross-sectional studies [20, 37], but other studies have not shown this effect [15]. In the present analyses, and in the FinnDiane type 1 diabetes study [15], lipid-modifying medication use was positively associated with telomere shortening. This likely represents confounding by indication as statin users usually have relatively adverse cardiometabolic profiles. The positive association with platelet counts and Shortened versus not Shortened ΔrTL is consistent with a cross-sectional study showing an inverse relationship between essential thrombocythaemia and telomere length [54]. The inverse association between serum bilirubin and telomere shortening is consistent with data from studies in the general population from NHANES [55] and in Gilbert’s syndrome [56]. Evidence suggests that mildly elevated serum bilirubin levels (> 10 mmol/L) reduce oxidative stress and exert anti-inflammatory effects in some metabolic and age-related diseases [57, 58]. An increased serum bilirubin in Gilbert’s syndrome (> 17.1 mmol/L) has been associated with longer telomeres than in non-affected individuals (concentration range 3–17.1 mmol/L) [56]. In addition, in 7818 adults from NHANES with a serum bilirubin < 17.1 mmol/L, there was a positive association between telomere length and serum bilirubin [55]. Common underlying mechanisms accelerated telomere shortening are known as increased oxidative stress and chronic inflammation, leading to cumulative DNA damage with impaired repair and reduced telomerase activity [8].

The various independent associations with ΔrTL are, therefore, largely consistent with the published literature. Regarding analytical validity, all DNA samples were analysed in triplicate on the same plate, using a well-validated protocol [28] with excellent intra- and inter-assay CVs. The longer telomeres at the second timepoint may reflect deterioration of stored DNA over time. However, there is evidence that telomere sequences deteriorate substantially in the first four years of storage then stabilise during long-term storage of up to 12 years [59]. Our baseline and Year-4 blood samples were stored at -80 °C for 12–15 years and 8–11 years, respectively, prior to rTL assay. Thus, the degree of telomere deterioration in samples from both time points should be similar and unlikely to be the contributor to the difference in rTL measurement using qPCR.

The strengths of the present study include the novelty of serial (two) telomere length measures by a robust assay, detailed subject characterisation of a community-based cohort of adults with type 2 diabetes and long follow-up. We measured rTL at two timepoints in a modestly large cohort, whilst previous reports were either single timepoint studies [13, 14, 30] or multiple timepoints examining the effects of lifestyle modification or weight loss in a smaller number of participants [71]. The present study had some limitations. We used relative rather than absolute telomere length but, due to the time-consuming and labour-intensive nature of the latter as comprehensively reviewed [8, 60], it is not readily applicable to large studies [8]. Observational studies can be affected by selection bias. Although FDS2 participants are well-characterised, covariates which may modulate telomere length including diet [61] and physical activity [62] were either self-reported or not recorded. The use of stepwise variable selection also has limitations, with the potential for biased coefficients and *P* values, and inflated model fit statistics.

## Conclusions

We found that, in community-based adults with type 2 diabetes, telomeres shorten over time in the minority. We found no association between baseline rTL or ΔrTL and the main incident diabetes complications. There was, however, a relatively modest association between ΔrTL and CVD death in multivariable analyses. Given that a relationship between baseline telomere length and the adverse outcomes of diabetes has been found in some [13, 14] but not all [63] studies, and in light of the changing epidemiology of micro- and macroangiopathy [42], measurement of rTL appears to add little to usual type 2 diabetes care.

## Electronic supplementary material

Below is the link to the electronic supplementary material.

Supplementary Material 1

Supplementary Material 2

Supplementary Material 3

Supplementary Material 4

Supplementary Material 5

Supplementary Material 6

Supplementary Material 7

## Data Availability

Some outcome data supporting the findings of this study are available from the Western Australian Department of Health, but restrictions apply to the availability of these data, which were used under strict conditions of confidentiality for the current study, and so are not publicly available. Data are however available from the authors upon reasonable request and with permission of Western Australian Department of Health.

## Abbreviations

ABSI: A body shape index
ACEi/ARB: Angiotensin converting enzyme inhibitors/angiotensin receptor blockers
CCI: Charlson comorbidity index
csHR: Cause-specific hazard ratios
DSPN: Distal symmetrical polyneuropathy
ESRD: End-stage renal disease
FDS2: Fremantle diabetes study phase II
HBG: Human β-globin
HMDC: Hospital morbidity data collection
hsCRP: High sensitivity C-reactive protein
IGT: Impaired glucose tolerance
LEA: Lower extremity amputation
MACE: Major adverse cardiovascular events
MI: Myocardial infarction
NHANES: National health and nutrition examination survey
OHA: Oral hypoglycaemic agent
PAD: Peripheral arterial disease
qPCR: Quantitative polymerase chain reaction
rTL: Relative telomere length
sdHR: Subdistribution hazard ratios
uACR: Urinary albumin:creatinine ratio
WA: Western Australia
WADLS: WA data linkage system
