# Supplementary material for: Determinants of temporal change in telomere length and its associations with chronic complications and mortality in type 2 diabetes: the Fremantle diabetes study phase II
Source: Cardiovasc Diabetol. 2025 Jul 3;24:267. doi: 10.1186/s12933-025-02832-3 (PMC12224854; doi:10.1186/s12933-025-02832-3)
Supplement: Supplementary file 1 — Supplementary Material 1 [file 12933_2025_2832_MOESM1_ESM.pdf]

Table S1. ΔrTL as continuous variable by change in potential risk factors and medication use between baseline and Year-4 in people with type 2 diabetes.

	n/N	Unadjusted regression coefficient	*P*-value	*P*-value*
Age (years)	819/819	-0.061	0.384	0.363
Male (%)	819/819	-4.722	**0.001**	**0.001**
Change in age (years)	819/819	-3.121	0.128	0.092
Change in diabetes duration (years)	819/819	-3.121	0.128	0.092
Change in smoking status:				
Stayed in same category	780/812	-0.443	0.907	0.803
Stopped	24/812	-3.701	0.397	0.473
Started	8/812	12.601	0.092	0.084
Change in diabetes treatment (%):				
Stayed in same category	635/808	1.159	0.520	0.485
De-intensified	23/808	5.808	0.192	0.127
Intensified	150/808	-2.353	0.216	0.166
Change in metformin use (%):				
Stayed in same category	674/807	1.318	0.509	0.547
Stopped	38/807	-0.293	0.933	0.895
Started	95/807	-1.620	0.481	0.435
Change in HbA_{1c} (%)	819/819	-0.069	0.905	0.812
Change in weight (kg)	819/819	-0.112	0.323	0.330
Change in BMI (kg/m^2)	819/819	-0.568	0.150	0.156
Change in ABSI (increase of 0.001 $m^{11/6} kg^{-2/3}$)	819/819	-0.098	0.574	0.650
Change in central obesity (by waist circumference; %)				
Stayed in same category	703/812	0.215	0.921	0.987
Centrally obese to normal	73/812	-1.013	0.695	0.805
Normal to centrally obese	36/812	1.365	0.704	0.711
Change in systolic blood pressure (mmHg)	819/819	0.013	0.676	0.795
Change in diastolic blood pressure (mmHg)	819/819	-0.025	0.635	0.281
Change in antihypertensive medication use (%):				
Stayed in same category	707/813	1.013	0.644	0.582
Stopped	30/813	-1.931	0.621	0.560
Started	76/813	-0.545	0.830	0.796
Change in ACEi/ARB use (%)				
Stayed in same category	659/813	2.673	0.151	0.168
Stopped	63/813	-1.669	0.545	0.546
Started	95/813	-2.901	0.206	0.230

Change in total serum cholesterol (mmol/L)	819/819	1.837	**0.006**	**0.005**
Change in serum HDL-cholesterol (mmol/L)	819/819	3.691	0.318	0.319
Change in serum triglycerides (mmol/L)	819/819	0.853	0.224	0.253
Change in lipid-lowering medication use (%)				
Stayed in same category	658/813	1.268	0.499	0.471
Stopped	60/813	-0.329	0.907	0.817
Started	95/813	-1.678	0.464	0.489
Change in statin use (%)				
Stayed in same category	650/818	1.139	0.531	0.496
Stopped	71/818	0.714	0.784	0.927
Started	97/818	-2.320	0.307	0.352
Change in fibrate use (%)				
Stayed in same category	785/813	-2.874	0.477	0.477
Stopped	4/813	-22.578	**0.032**	**0.029**
Started	24/813	7.195	0.098	0.095
Change in eGFR (CKD-EPI) (ml/min/1.73m^2)	819/819	0.024	0.737	0.580
Change in uACR (mg/mmol)	819/819	0.001	0.946	0.962

P-values derived from linear regression with ΔrTL (continuous variable) as the dependent variable.

**P*-value adjusted for age and sex.
