# Supplementary material for: Determinants of temporal change in telomere length and its associations with chronic complications and mortality in type 2 diabetes: the Fremantle diabetes study phase II
Source: Cardiovasc Diabetol. 2025 Jul 3;24:267. doi: 10.1186/s12933-025-02832-3 (PMC12224854; doi:10.1186/s12933-025-02832-3)
Supplement: Supplementary file 2 — Supplementary Material 2 [file 12933_2025_2832_MOESM2_ESM.pdf]

Table S2. Baseline rTL in participants by ΔrTL category.

ΔrTL category	Number (%)	Baseline rTL		Comparison vs Lengthened	
		Mean (SD)	Minimum/Maximum	Mean difference (SD)	Bonferroni-adjusted *P*-value
Shortened	209 (25.5%)	0.211 (0.89)	-2.289/2.769	0.986* (0.06)	<0.001
Unchanged	86 (10.5%)	0.046 (0.65)	-1.705/1.657	0.821* (0.09)	<0.001
Lengthened	524 (64.0%)	-0.774 (0.83)	-4.676/1.136	-	-
Total	819	-0.436 (0.94)	-4.676/2.769	-	-

*The mean difference is significant at the 0.05 level.
