# Supplementary material for: Determinants of temporal change in telomere length and its associations with chronic complications and mortality in type 2 diabetes: the Fremantle diabetes study phase II
Source: Cardiovasc Diabetol. 2025 Jul 3;24:267. doi: 10.1186/s12933-025-02832-3 (PMC12224854; doi:10.1186/s12933-025-02832-3)
Supplement: Supplementary file 3 — Supplementary Material 3 [file 12933_2025_2832_MOESM3_ESM.pdf]

Table S3. Baseline characteristics by ΔrTL Shortened vs Not Shortened over 4 years in people with type 2 diabetes.

	Shortened	Not Shortened	*P*-value
Number (%)	209 (25.5)	610 (74.5)	
Age (years)	66.2±10.5	65.5±10.4	0.452
Male (%)	61.7	51.0	0.008
Ethnic background (%):			0.337
Anglo-Celt	61.7	57.9	
Southern European	11.0	11.5	
Other European	5.7	8.4	
Asian	2.4	5.1	
Aboriginal	3.3	15.2	
Mixed/other	15.8	2.0	
Age at diabetes diagnosis (years)	56.2±11.1	56.1±11.5	0.934
Diabetes duration (years)	8.0 [3.0-15.4]	8.0 [2.0-15.0]	0.257
Smoking status (%)			0.014
Never	34.4	45.5	
Ex	58.4	47.0	
Current	7.2	7.6	
Alcohol consumption (standard drinks/day)	0.3 [0-1.5]	0.1 [0-1.2]	0.229
Fasting serum glucose (mmol/L)	7.1 [6.1-8.7]	7.0 [6.2-8.4]	0.812
HbA_{1c} (%)	6.8 [6.3-7.6]	6.7 [6.1-7.5]	0.204
Diabetes treatment (%)			0.511
Diet	24.9	29.2	
Oral hypoglycemic agents (OHAs)/non-insulin injectables	55.0	51.6	
Insulin only	5.3	3.8	
Insulin+OHAs/non-insulin injectables	14.8	15.4	
Taking metformin (%)	65.1	62.2	0.507
BMI (kg/m^2)	31.8±5.7	31.1±5.8	0.143
ABSI ($m^{11/6}$ $kg^{-2/3}$)	0.082±0.005	0.081±0.005	0.044
Central obesity (by waist circumference; %)	77.4	69.7	0.033
Systolic blood pressure (mmHg)	146±22	145±21	0.742
Diastolic blood pressure (mmHg)	80±11	80±12	0.827
Taking antihypertensive medication (%)	77.0	74.1	0.460
Taking ACEi/ARB (%)	66.0	66.6	0.932
Total serum cholesterol (mmol/L)	4.3±1.3	4.4±1.1	0.849
Serum HDL-cholesterol (mmol/L)	1.26±0.36	1.24±0.30	0.357
Serum triglycerides (mmol/L)	1.5 (0.9-2.4)	1.5 (0.9-2.4)	0.895
Taking lipid-modifying medication (%)	77.0	68.4	0.018

Taking fibrates (%)	3.3	2.0	0.286
hsCRP (mg/L)	2.3 (0.7-7.2)	2.1 (0.7-6.3)	0.418
Taking aspirin (%)	40.9	37.8	0.458
Cerebrovascular disease (%)	8.6	6.4	0.273
Coronary heart disease (%)	26.8	26.4	0.928
Peripheral arterial disease (%)	17.8	19.7	0.611
Distal symmetrical polyneuropathy (2-point; %)	36.1	36.6	0.934
Any retinopathy (%)	35.0	33.9	0.799
eGFR (CKD-EPI) category (%)			0.354
≥90 ml/min/1.73m^2	36.2	39.8	
60-89 ml/min/1.73m^2	50.7	46.5	
45-59 ml/min/1.73m^2	5.8	8.6	
30-44 ml/min/1.73m^2	5.3	4.1	
<30 ml/min/1.73m^2	1.9	1.0	
Urinary albumin:creatinine ratio (mg/mmol)	3.1 (0.9-11.0)	2.9 (0.9-9.4)	0.394
Platelets (x10^9/L)	252 (194-329)	244 (188-317)	0.105
Serum albumin (g/L)	44±3	44±3	0.798
Gamma-glutamyl transferase (U/L)	33 (16-67)	29 (15-57)	0.058
Bilirubin (μmol/L)	9.3 (6.4-13.4)	10.1 (6.8-15.1)	0.005
Alpha-2 macroglobulin (g/L)	2.03 (1.43-2.88)	2.06 (1.47-2.90)	0.543
Haptoglobulin (g/L)	1.55±0.57	1.51±0.55	0.387

Shortened ΔrTL was defined as ΔrTL <-2.69% and Not Shortened as ΔrTL >-2.69%.
