# Supplementary material for: Determinants of temporal change in telomere length and its associations with chronic complications and mortality in type 2 diabetes: the Fremantle diabetes study phase II
Source: Cardiovasc Diabetol. 2025 Jul 3;24:267. doi: 10.1186/s12933-025-02832-3 (PMC12224854; doi:10.1186/s12933-025-02832-3)
Supplement: Supplementary file 4 — Supplementary Material 4 [file 12933_2025_2832_MOESM4_ESM.pdf]

Table S4. Baseline/Year-4 rTL and ΔrTL by all-cause mortality to end-2021 in people with type 2 diabetes.

		Alive	Deceased	*P*-value
Number (%)		606 (74.0)	213 (26.0)	
Baseline rTL		-0.40±0.94	-0.55±0.97	0.043
Year-4 rTL		-0.004±1.32	-0.30±1.11	0.004
ΔrTL (continuous)		9.2±21.1	6.2±20.6	0.078
ΔrTL categories (%):				0.267
	Shortened	26.7	32.4	
	Unchanged	5.4	5.6	
	Lengthened	67.8	62.0	
