# Supplementary material for: Determinants of temporal change in telomere length and its associations with chronic complications and mortality in type 2 diabetes: the Fremantle diabetes study phase II
Source: Cardiovasc Diabetol. 2025 Jul 3;24:267. doi: 10.1186/s12933-025-02832-3 (PMC12224854; doi:10.1186/s12933-025-02832-3)
Supplement: Supplementary file 5 — Supplementary Material 5 [file 12933_2025_2832_MOESM5_ESM.pdf]

Table S5. Competing risk regression models of time to CVD mortality with measures of rTL (added separately) as the main variable of interest. Data are subdistribution hazard ratios (sdHR) and 95% confidence intervals (CI).

	sdHR (95% CI)	*P*-value	sdHR (95% CI)	*P*-value	sdHR (95% CI)	*P*-value	sdHR (95% CI)	*P*-value
Baseline rTL (increase of 1)	0.90 (0.69, 1.18)	0.452						
Year-4 rTL (increase of 1)			0.86 (0.76, 0.97)	0.015				
ΔrTL (increase of 1)					0.99 (0.98, 1.004)	0.197		
ΔrTL categories:								
Unchanged							1.00	
Shortened							1.02 (0.48, 2.18)	0.963
Lengthened							0.81 (0.41, 1.64)	0.563

All models are adjusted for age, insulin use, heart rate, ln(NT-proBNP), peripheral arterial disease, retinopathy, and Charson’s Comorbidity Index.
