# Supplementary material for: Determinants of temporal change in telomere length and its associations with chronic complications and mortality in type 2 diabetes: the Fremantle diabetes study phase II
Source: Cardiovasc Diabetol. 2025 Jul 3;24:267. doi: 10.1186/s12933-025-02832-3 (PMC12224854; doi:10.1186/s12933-025-02832-3)
Supplement: Supplementary file 6 — Supplementary Material 6 [file 12933_2025_2832_MOESM6_ESM.pdf]

Table S6. Cox proportional regression models of time to CVD mortality with measures of rTL (added separately) as the main variable of interest, Year-4 as baseline and Year-4 variables considered for entry. Data are cause-specific hazard ratios (csHR) and 95% confidence intervals (CI).

	csHR (95% CI)	*P*-value	csHR (95% CI)	*P*-value	csHR (95% CI)	*P*-value
Year-4 rTL (increase of 1)	0.89 (0.81, 0.99)	0.036				
ΔrTL (increase of 1)			0.99 (0.98, 1.002)	0.109		
ΔrTL categories:						
Unchanged					1.00	
Shortened					0.93 (0.43, 2.00)	0.852
Lengthened					0.79 (0.39, 1.61)	0.521

All models are adjusted for Year-4 age, insulin use, peripheral arterial disease, eGFR (CKD-EPI) <30 ml/min/1.73m^2, and Charson's Comorbidity Index.
