# Supplementary material for: Determinants of temporal change in telomere length and its associations with chronic complications and mortality in type 2 diabetes: the Fremantle diabetes study phase II
Source: Cardiovasc Diabetol. 2025 Jul 3;24:267. doi: 10.1186/s12933-025-02832-3 (PMC12224854; doi:10.1186/s12933-025-02832-3)
Supplement: Supplementary file 7 — Supplementary Material 7 [file 12933_2025_2832_MOESM7_ESM.pdf]

Table S7. Competing risk regression models of time to cardiovascular disease mortality with measures of rTL (added separately) as the main variable of interest, Year-4 as baseline and Year-4 variables considered for entry. Data are subdistribution hazard ratios (sdHR) and 95% confidence intervals (CI).

	sdHR (95% CI)	*P*-value	sdHR (95% CI)	*P*-value	sdHR (95% CI)	*P*-value
Year-4 rTL (increase of 1)	0.88 (0.80, 0.97)	0.013				
ΔrTL (increase of 1)			0.99 (0.98, 0.9997)	0.045		
ΔrTL categories:						
Unchanged					1.00	
Shortened					0.87 (0.41, 1.83)	0.713
Lengthened					0.68 (0.34, 1.36)	0.271

All models are adjusted for Year-4 age, insulin use, peripheral arterial disease, eGFR (CKD-EPI) <30 ml/min/1.73m^2, and Charson's Comorbidity Index.
